# Verrucomicrobia of the Family *Chthoniobacteraceae* Participate in Xylan Degradation in Boreal Peat Soils

**DOI:** 10.3390/microorganisms12112271

**Published:** 2024-11-08

**Authors:** Andrey L. Rakitin, Irina S. Kulichevskaya, Alexey V. Beletsky, Andrey V. Mardanov, Svetlana N. Dedysh, Nikolai V. Ravin

**Affiliations:** 1 Institute of Bioengineering, Research Center of Biotechnology, Russian Academy of Sciences, Moscow 119071, Russia; 2 Winogradsky Institute of Microbiology, Research Center of Biotechnology, Russian Academy of Sciences, Moscow 119071, Russiadedysh@mail.ru (S.N.D.)

**Keywords:** *Verrucomicrobiota*, *Chthoniobacteraceae*, peatland, xylan degradation, metagenome

## Abstract

The phylum *Verrucomicrobiota* is one of the main groups of soil prokaryotes, which remains poorly represented by cultivated organisms. The major recognized role of *Verrucomicrobiota* in soils is the degradation of plant-derived organic matter. These bacteria are particularly abundant in peatlands, where xylan-type hemicelluloses represent one of the most actively decomposed peat constituents. The aim of this work was to characterize the microorganisms capable of hydrolyzing xylan under the anoxic conditions typical of peatland soils. The laboratory incubation of peat samples with xylan resulted in the pronounced enrichment of several phylotypes affiliated with the *Verrucomicrobiota*, *Firmicutes*, and *Alphaproteobacteria*. Sequencing of the metagenome of the enrichment culture allowed us to recover high-quality metagenome-assembled genomes (MAGs) assigned to the genera *Caproiciproducens, Clostridium*, *Bacillus* (*Firmicutes*), *and Rhizomicrobium* (*Alphaproteobacteria*), *Cellulomonas* (*Actinobacteriota*) and the uncultured genus-level lineage of the family *Chthoniobacteraceae* (*Verrucomicrobiota*). The latter bacterium, designated “*Candidatus* Chthoniomicrobium xylanophilum” SH-KS-3, dominated in the metagenome and its MAG was assembled as a complete closed chromosome. An analysis of the SH-KS-3 genome revealed potential endo-1,4-beta-xylanases, as well as xylan beta-1,4-xylosidases and other enzymes involved in xylan utilization. A genome analysis revealed the absence of aerobic respiration and predicted chemoheterotrophic metabolism with the capacity to utilize various carbohydrates, including cellulose, and to perform fermentation or nitrate reduction. An analysis of other MAGs suggested that *Clostridium* and *Rhizomicrobium* could play the role of primary xylan degraders while other community members probably took advantage of the availability of xylo-oligosaccharides and xylose or utilized low molecular weight organics.

## 1. Introduction

The phylum *Verrucomicrobiota* is one of the globally distributed and highly diverse lineages of the domain *Bacteria*, which remains poorly represented by cultivated and phenotypically characterized organisms. Being initially discovered in a freshwater habitat [1], members of this phylum were later detected in various marine and freshwater ecosystems [2,3], the digestive tract of insects [4,5] and animals [6], and various soils around the world. The latter represents one of the major habitats where verrucomicrobia are particularly abundant and diverse [7,8,9]. Thus, members of the *Verrucomicrobiota* were estimated to comprise between 1.2 and 10.9% of the total bacteria in soil, while on average their relative abundance assessed by 16S rRNA gene amplicon sequencing in soil habitats was about 5% of the total bacterial diversity [7]. According to the high proportion of *Verrucomicrobiota*-affiliated sequences in 16S rRNA gene libraries, up to 9.8%, these bacteria are among the metabolically active members of the soil microbial communities [7,10]. 

Most of the currently characterized verrucomicrobia are aerobic or anaerobic chemoorganotrophs, which utilize a wide range of sugars and sugar polymers, including the key components of the plant biomass, such as cellulose and xylan. The only known exception is members of the family *Methylacidiphilaceae*, who possess particulate methane monooxygenase enzymes and are autotrophic methanotrophs [11]. These verrucomicrobial methanotrophs, however, are restricted to extremely acidic geothermal habitats and are rarely detected in other ecosystems. The major recognized environmental role of verrucomicrobia, therefore, is the degradation of plant-derived organic matter. 

One of the soil habitats where *Verrucomicrobiota* constitutes a significant part of the bacterial community is peatlands [12,13]. Peatlands cover about 4.16 × 10^6^ km^2^ worldwide [14] and serve as a persistent sink for CO_2_ and an important carbon store [15]. Carbon is stored in the form of dead organic residues, whose composition depends on the peatland type and the indigenous plant community. Among the recognized peatland types, raised bogs, which are fed solely by precipitation, and eutrophic fens, which are fed mainly by ground water, represent two of the most contrasting and widely distributed types of peatlands. Bogs are highly acidic (pH values are around 4.0) and nutrient-poor, and are dominated by *Sphagnum* mosses. Fens are near-neutral, more nutrient-rich than bogs, and are covered by sedges and grasses. Due to environmental factors as well as differences in litter quality, decomposition processes in fens have been shown to proceed more quickly than in bogs [16]. As shown in field experiments with litter bags, the *Carex*-derived phytomass decomposed significantly faster than the *Sphagnum* litter [17], which is one of the reasons why C turnover rates in fens are much faster than those in bogs. The variation in the rate of carbon recycling in different types of peatlands is also attributed to abiotic factors, such as pH and the availability of mineral nutrients, and the microbial diversity of these habitats, which increases from nutrient-poor bogs to rich fens [18,19]. Thus, it has been speculated that relatively fast C turnover rates in fens are determined, at least in part, by the increased abundance of *Bacteroidota* [18,19], whose hydrolytic capabilities are well-recognized.

A recent organochemical analysis of peat decomposition revealed the preferential degradation of specific side-chain linkages of xylan-type hemicelluloses in the peat from surface layers (0–40 cm), while the xylan backbone, galactomannan-type hemicelluloses, and cellulose were more resistant to degradation [20]. Thus, xylan-type hemicelluloses represent one of the most actively decomposed peat constituents. Xylan is the second most abundant polysaccharide of the plant biomass after cellulose [21,22,23]. Xylan consists of a linear chain of β-1,4-linked xylopyranosyl residues that are often substituted with side groups such as arabinose, acetyl, glucuronic acids, ferulic acid, and p-coumaric acid [21]. Due to the structural complexity of xylans, different types of enzymes are required for its hydrolysis. Endo-1,4-β-xylanases (EC 3.2.1.8) randomly cleave the β-1,4 linkages between the xylopyranosyl residues in the main chain of xylan. Xylan 1,4-β-D-xylosidase (EC 3.2.1.37) catalyzes the hydrolysis of non-reducing end xylose residues from xylooligosaccharides generated by endoxylanase. A variety of branch point-degrading enzymes include α-L-arabinofuranosidase (EC 3.2.1.55), acetyl xylan esterase (EC 3.1.1.72), ferulic acid esterase (EC 3.1.1.73), and α-D-glucuronidase (EC 3.2.1.139) [21]. 

The knowledge of xylan-degrading microorganisms in fens remains limited. We are not aware of any specialized study that would address this subject. The potential participation of peat-inhabiting fungi [18], *Firmicutes* [18], or *Bacteroidota* [18,19] with characterized xylanolytic capabilities cannot be excluded. However, the contribution of other, as-yet-uncharacterized microbes may also be substantial. Peat-inhabiting verrucomicrobia may well-represent one of these groups. As shown in the recent comparative diversity analysis of *Verrucomicrobiota* in six boreal fens, the most abundant peat-inhabiting representatives were members of the families *Pedosphaeraceae*, *Opitutaceae,* and *Chtoniobacteraceae* [24]. Notably, strains representing the type genera of these families, i.e., *Pedosphaera*, *Opitutus*, and *Chthoniobacter*, were isolated from soil habitats and were characterized as aerobic and anaerobic heterotrophic bacteria with high hydrolytic potential [25,26,27]. The growth substrates of these bacteria include a wide range of monosaccharides, disaccharides, and polysaccharides, including xylan and pectin. *Chthoniobacter flavus* was also reported as being able to grow on cellulose [26]. This work, therefore, was undertaken in order to obtain a deeper knowledge of the hydrolytic potential of fen-inhabiting verrucomicrobia, namely their ability to degrade xylan, one of the key biopolymers in peatlands.

## 2. Materials and Methods

### 2.1. Source of Isolation

Peat soil samples were collected from two eutrophic fens, Shichengskoe (59°56′31.6″ N, 41°15′53.5″ E) and Charozerskoe (60°30′42.3″ N, 38°39′04.6″ E), located in the Vologda region in the North European Russia. The samples were taken at the oxic–anoxic peat interface, i.e., at a depth of 10 to 12 cm in Shichengskoe fen and at a depth of 1 to 3 cm in Charozerskoe fen. The physical and chemical characteristics of peat water were typical for eutrophic fens (Table 1). Plant cover in the fen Shichengskoe was represented by the association of *Equisetum palustre* and *Sphagnum warnstorfii*, while dominant plant species in the fen Charozerskoe were *Carex lasiocarpa* and *Campylium stellatum*. Detailed characteristics of the sampling sites were reported previously [12,13].

### 2.2. Anaerobic Enrichment Cultures with Xylan as a Growth Substrate

The collected peat samples were used to obtain the enrichment cultures of microorganisms that participate in the hydrolysis of xylan, one of the key biopolymers of wetland ecosystems. The peat sample was crushed with sterile scissors to fragments of about 5 mm in size and mixed thoroughly. To set up enrichment cultures, 2 g of homogenized peat and 30 mL of native peat water were placed into 160 mL screw-cap glass flasks. 0.5 g of xylan (Sigma, Deisenhofen, Germany) was added to each incubation, after which the flasks were hermetically closed with butyl rubber septa and flushed with a mixture of N_2_:CO_2_ (80:20) to create anaerobic conditions. The flasks were then incubated at room temperature (20–22 °C) for 8 weeks. Monitoring of the microbial community developing in the xylan-supplemented peat suspension was carried out once a week using phase-contrast microscopy via a Zeiss Axioplan 2 microscope (Jena, Germany).

After 8 weeks of incubation, 5 mL aliquots of the obtained cultures were transferred to Hungate tubes and mixed with an equal volume of sterile nutrient medium of the following composition (g/L): xylan, 2; NH_4_Cl, 0.5; MgCl_2_ × 6H_2_O, 0.2; CaCl_2_, 0.15; KH_2_PO_4_, 0.15; NaHCO_3_, 0.5; yeast extract, 0.1; trace element solution 1.0 mL; vitamin solution, 1.0 mL. To create anaerobic conditions, the tubes were flushed with a mixture of N_2_:CO_2_ (80:20). Incubation was carried out at room temperature (20–22 °C) for 4 weeks. Analysis of the microbial community composition in the resulting enrichment cultures for the presence of verrucomicrobia was carried out via fluorescence in situ hybridization (FISH) as described below.

### 2.3. FISH-Based Analysis of Cultures for the Presence of Verrucomicrobiota

Cells of the enrichment cultures were harvested via centrifugation and resuspended in 0.5 mL of phosphate-buffered saline (PBS) containing, in grams per liter, NaCl, 8.0; KCl, 0.2; Na_2_HPO_4_, 1.44; and NaH_2_PO_4_, 0.2 (pH 7.0). The cell suspension was mixed with 1.5 mL of 4% (*w/v*) freshly prepared paraformaldehyde solution (Sigma, Deisenhofen, Germany) and fixed for 1 h at room temperature. The cells were then collected by centrifugation (6600× *g* for 1 min) and washed twice with PBS to ensure the removal of paraformaldehyde. The resulting cell pellet was resuspended in 0.5 mL of 50% ethanol–PBS (*v/v*). The Cy-labeled oligonucleotide probe EUBIII, specific for most representatives of the phylum Verrucomicrobiota [28], was purchased from Syntol (Moscow, Russia). Hybridization was performed on gelatin-coated (0.1%, *w/v*) and dried Teflon-laminated slides. The fixed cell samples were applied to these wells and hybridized for 1.5 h at 46 °C to the corresponding fluorescent probe with 20% (*v*/*v*) formamide in hybridization buffer. The specimens were examined with a ZeissAxioplan2 microscope (Zeiss, Jena, Germany) equipped with the Zeiss Filter No 02 for the Cy3-labeled probe. 

### 2.4. DNA Isolation and 16S rRNA Gene Profiling

The cells of the enrichment cultures were harvested via centrifugation and resuspended total genomic DNA from enrichment cultures was isolated using a Power Soil DNA isolation kit (MO BIO Laboratories, Carlsbad, CA, USA), and stored at −20 °C.

The 16S rRNA gene fragments comprising the V3–V4 variable regions were amplified by PCR using the universal prokaryotic primers 341F (50-CCTAYG GGDBGCWSCAG) and 806R (50-GGA CTA CNVGGG THTCTAAT) [29]. The obtained fragments were bar-coded using the Nextera XT Index Kit v. 2 (Illumina, San Diego, CA, USA) mixed and sequenced on Illumina MiSeq (2 × 300 nt reads). Pairwise overlapping reads were merged using FLASH v.1.2.11 [30].

All the sequences were clustered into operational taxonomic units (OTUs) at 97% identity using the USEARCH v.11 program [31]. Low quality reads were removed prior to clustering; chimeric sequences and singletons were removed during clustering by the USEARCH algorithms. To calculate the OTU abundances, all the reads obtained for a given sample were mapped to OTU sequences at a 97% global identity threshold by USEARCH. The taxonomic assignment of OTUs was performed by searching against the SILVA v.138 rRNA sequence database using the VSEARCH v. 2.14.1 algorithm [32].

### 2.5. Metagenome Sequencing and Assembly of MAGs

The metagenome sequencing of total DNA isolated from enrichment cultures was performed using Illumina and Oxford Nanopore platforms. The library for Illumina sequencing was prepared using the NEBNext Ultra II DNA library preparation kit (New England Biolabs, Ipswich, MA, USA) and sequenced on an Illumina MiSeq instrument in paired-end (2 × 300 bp) mode. The sequencing generated 1,644,118 read pairs (~990 Mb). The trimming of low-quality sequences (Q < 30) was performed using Sickle v.1.33. For nanopore sequencing, the ligation sequencing kit 1D was used for sample preparation. Sequencing was performed in FLOMIN110 cells using the MinION instrument (Oxford Nanopore Technologies, Oxford, UK) and generated 1,664,623 reads (~3.5 Gb).

MinION reads were assembled into contigs using Flye v. 2.9 [33] in a metagenome mode (-meta). The consensus sequence of the obtained contigs was polished twice using Illumina reads and NextPolish v.1.4.1 [34]. MAGs were assembled using three programs: MaxBin v.2.2.7 [35], CONCOCT v.1.0.0 [36] and MetaBAT v.2.2.15 [37]. The assembly results were improved using the DAS Tool v.1.1.4 [38]. The obtained MAGs were taxonomically assigned, employing the GTDB Toolkit (GTDB-Tk) v.2.0.0 [39] and the Genome Taxonomy Database (GTDB) [40]. The completeness and redundancy (contamination) of the obtained MAGs were assessed using CheckM v.1.1.3 [41].

### 2.6. Analysis and Annotation of MAGs

The prediction of genes and their annotation were performed using the National Center for Biotechnology Information (NCBI) Prokaryotic Genome Annotation Pipeline v.6.5 [42]. The N-terminal signal peptides were predicted by Signal P v.5.0 [43].

The average amino acid identity (AAI) and average nucleotide identity (ANI) between genomes were calculated using tools from the Enveomics Collection [44].

For phylogenetic analysis based on genome sequences, GTDB-Tk v.2.0.0 was employed to find 120 single-copy marker genes and to build multiple alignments of concatenated amino acid sequences. A maximum likelihood tree was built using PhyML v. 3.3 [45] and branch support values were calculated via the approximate Bayes method. Default settings were used for all the software unless otherwise noted.

The Distilled and Refined Annotation of Metabolism (DRAM) tool [46] was used for the prediction and comparative analysis of the metabolic capabilities encoded by the genomes.

## 3. Results

### 3.1. Microbial Community Response to Incubation with Xylan

**Figure 1:**
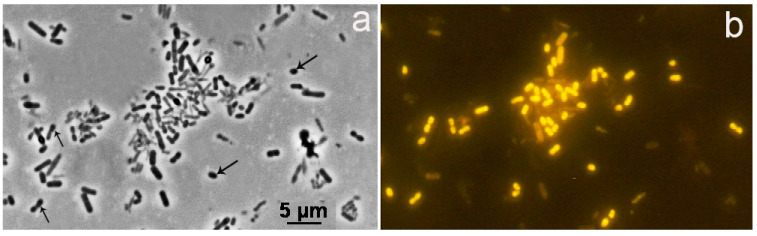
Specific detection of verrucomicrobia in an enrichment culture of an anaerobic xylanolytic microbial community obtained from the peat of the boreal fen, Shichengskoe. (**a**) Phase contrast microscopy. (**b**) Epifluorescent microphotographs of cell hybridization with Cy3-labeled probe EUBIII. The scale bar applies to both images.

To enrich the peat-inhabiting microorganisms involved in xylan degradation, peat suspensions from the fens Shichengskoe and Charozerskoe were incubated with xylan under anaerobic conditions. The microscopic analysis of primary enrichment cultures showed that by the end of the 8-week incubation, the composition of microbial communities was diverse and, besides the common spore-forming cell morphotypes of the well-studied bacteria of the phylum *Firmicutes* (*Bacillota*), included a number of cells represented by short rods or ovoids. These enrichment cultures were used to inoculate a mineral medium supplemented with xylan and the incubation was continued. By the end of the 4-week incubation, the resulting secondary enrichment cultures contained the bacteria of 3–4 morphotypes, with short (0.8–1.5 µm) rod-shaped or ovoid cells being clearly dominant. Notably, these cells hybridized with the probe EUBIII, specific for most members of *Verrucomicrobiota,* as revealed by the strong probe-conferred signal (Figure 1).

**Figure 2:**
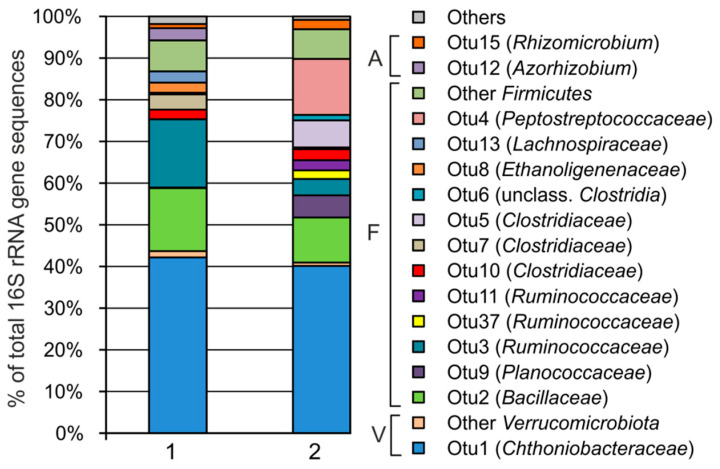
Microbial community composition of xylan-supplied enrichment cultures from fens Shichengskoe (1) and Charozerskoe (2) according to 16S rRNA gene profiling. OTUs with a relative abundance of more than 1% in at least one enrichment are shown. Abbreviations: A, *Alphaproteobacteria*; F, *Firmicutes*; V, *Verrucomicrobiota*.

The composition of these xylan-supplied enrichments was analyzed by 16S rRNA gene profiling and compared to original microbial communities detected in the same peat samples, reported previously [13]. The strongest positive response to xylan availability was detected for the members of the phyla *Firmicutes* and *Verrucomicrobiota* (Figure 2). Both enrichments were dominated by a small number of OTUs representing *Firmicutes* (50.6% and 56.0% of all the 16S rRNA gene sequences in enrichments from fens Shichengskoe and Charozerskoe, respectively) and *Verrucomicrobiota* (43.7% and 40.9%). In the original peat microbiomes, these phyla were present in minor fractions: *Verrucomicrobiota* accounted for about 6 and 3% of the communities in Shichengskoe and Charozerskoe fens, respectively, while the fractions of *Firmicutes* were less than 0.1%.

The absolute majority of *Verrucomicrobiota* were represented by a single OTU1, the shares of which in the enrichment cultures from Shichengskoe and Charozerskoe fens were 42.2% and 40.1%, respectively. In the original peat samples, the relative abundance of this OTU was less than 0.1%. Although *Firmicutes* were more diverse, only 12 OTUs accounted for more than 1% of the microbiome in at least one of the two enrichments. The most numerous phylotypes were the bacilli of the families *Bacillaceae* and *Planococcaceae*, as well as the clostridia of the families *Ruminococcaceae*, *Clostridiaceae*, *Ethanoligenenaceae*, *Peptostreptococcaceae* and *Lachnospiraceae* (according to the SILVA taxonomy). A total of 9 of the 12 OTUs were detected in both of the enrichment cultures, OTU13—only in the culture from the Shichengskoe fen, OTU4 and OTU5—only in the culture from the Charozerskoe fen. Two OTUs of *Alphaproteobacteria* of the genera *Azorhizobium* (2.9% and 0.01%) and *Rhizomicrobium* (1.0% and 2.3%) were also detected among ones enriched on xylan.

Thus, as a result of the growth with xylan under anaerobic conditions, a small number of *Verrucomicrobiota*, *Firmicutes* and *Alphaproteobacteria* phylotypes were selected because of their ability to hydrolyze xylan and/or utilize the products of its hydrolysis. To characterize the xylanolytic abilities of the members of this selected community, a metagenomic analysis of the enrichment culture from the Shichengskoe fen was carried out.

### 3.2. Assembly and Phylogenetic Placement of MAGs

Sequencing of the metagenome of the enrichment culture from the Shichengskoe fen using Illumina MiSeq and Oxford Nanopore techniques allowed us to recover six MAGs with more than 90% completeness and less than 5% contamination, as estimated by CheckM (Table 2). In total, these MAGs accounted for about 72% of all the metagenome sequences. The taxonomic identification of these MAGs based on the searches against GTDB revealed the same most abundant phylotypes that were found according to the 16S rRNA gene profiling.

**Figure 3:**
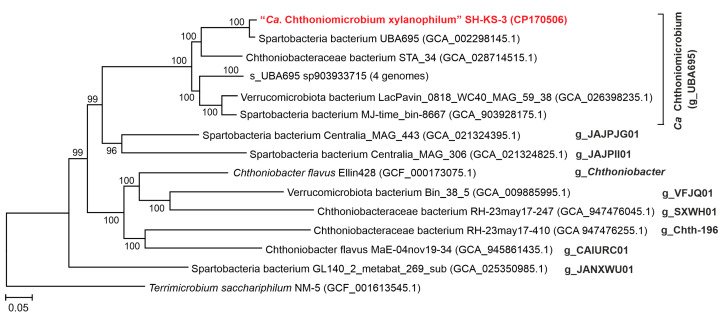
Phylogenomic placement of the SH-KS-3 genome in the maximum-likelihood protein phylogeny of *Chthoniobacteraceae*. The level of support for internal branches was assessed using the Bayesian test in PhyML. Taxonomy is shown according to the GTDB release RS220 (s, species; g, genus). The genome of *Terrimicrobium sacchariphilum* representing a sister family *Terrimicrobiaceae* of the order *Chthoniobacterales* was used to root the tree.

A single verrucomicrobial MAG-designated SH-KS-3 was assembled into a circular 5,624,388 bp long contig and assigned to the candidate genus UBA695 of the family *Chthoniobacteraceae*. This genotype accounted for 58.5% of the whole metagenome. To characterize the phylogenetic position of SH-KS-3 bacterium, a phylogenetic tree based on the concatenated sequences of 180 conserved marker genes, including SH-KS-3 MAG, all the other MAGs from UBA695, and representative genomes from all the other genus-level lineages of *Chthoniobacteraceae* was constructed (Figure 3). 

All the genera recognized by the GTDB within the *Chthoniobacteraceae* were represented by distinct monophyletic branches. The average amino acid sequence identity (AAI) between the SH-KS-3 genome and the genome of the only cultured member of *Chthoniobacteraceae*, *C. flavus*, was 52%, which is below the proposed genera delineation threshold of 65% [47] and supports the assignment of SH-KS-3 to a genus distinct from *Chthoniobacter*. Considering MAGs, the closest relative of SH-KS-3 was Spartobacteria bacterium, UBA695 (Figure 3), with an 89.9% AAI genome-to-genome distance; these genomes therefore represented different species of the candidate genus, UBA695.

Three MAGs were assigned to the phylum *Firmicutes*, and the genera *Caproiciproducens*, *Clostridium*, and *Bacillus*. These genomes corresponded to abundant OTUs 3, 7 and 2, revealed by 16S rRNA gene profiling. Surprisingly, altogether these MAGs accumulated less than 10% of the whole metagenome. Probably, a high number of rRNA operons in these genomes (e.g., five copies were found in the complete genome of *Caproiciproducens* SH-KS-11 versus two copies in *Chthoniobacteraceae* MAG SH-KS-3) resulted in the overestimation of their abundance via 16S rRNA gene profiling. One obtained MAG was assigned to the genus *Rhizomicrobium* and one represented the genus *Cellulomonas* of the phylum *Actinobacteriota*.

### 3.3. Genome-Based Analysis of Carbohydrate Utilization Pathways in Chthoniobacteraceae SH-KS-3 Bacterium

Taking into account that *Chthoniobacteraceae* SH-KS-3 dominated xylan enrichment and therefore could play a key role in xylan hydrolysis and that this genome is only the second complete genome for the family *Chthoniobacteraceae*, the metabolic potential of the SH-KS-3 bacterium were analyzed in detail. As a result of SH-KS-3 genome annotation, 4664 potential protein-coding genes were predicted, and the functions of only half of them were tentatively assigned. Two copies of the rRNA operon (16S-23S-5S), 49 tRNA genes, and a single CRISPR locus with 29 spacer-repeat units were predicted.

The finding of genes of the rod-shaped determining proteins MreBC, RodA, and peptidoglycan D,D-transpeptidase MrdA [48] indicates that the cells of the SH-KS-3 bacterium are rod-shaped, which is consistent with microscopic observations (Figure 1). Genes for flagellar machinery were absent but genes encoding the type 4 pili for twitching motility were found. The type 4 pili enable the attachment of the cells to insoluble substrates [49], and their presence is consistent with the predicted ability of SH-KS-3 to hydrolyze xylan.

A search for carbohydrate-active enzymes (CAZy) [50] revealed 371 domains, including ones for glycoside hydrolases (189), glycoside transferases (79), carbohydrate esterases (56), polysaccharide lyases (15) and carbohydrate-binding modules (28). Xylanases were found in several glycoside hydrolases (GH) families: 5, 7, 8, 10, 11, 26, 30, and 43 [21]. Endo-1,4-β-xylanase of the GH11 family, comprising true xylanases that are strict to xylan [21] were not identified. Eight enzymes were predicted to contain GH5 domain typically found in endoglucanases, but endo-β-1,4-xylanase activities were also described for this family; six of the GH5 enzymes contained N-terminal secretion signal peptide suggesting their extracellular operation. Endo-β-1,4-xylanase and endo-β-1,4-glucanase activities were also reported for GH8; one such enzyme carrying an N-terminal signal peptide was predicted for the SH-KS-3 bacterium. A single GH26 family hydrolase containing COG4124 domain of β-mannanase was unlikely involved in xylan hydrolysis, as well as two GH30 enzymes lacking N-terminal signal peptides. A single GH43 enzyme containing a secretion signal belonged to the GH43_22 subfamily where xylan β-1,4-xylosidase, rather than endoxylanase activity, was described. In search of potential endoxylanases, we also identified three proteins containing carbohydrate-binding domain CBM9, enabling binding to xylan. All of them contained N-terminal secretion signals. The first enzyme additionally contained GH10 catalytic domain, which is typically found in endo-β-1,4-xylanases and endo-β-1,4-glucanase; it may be directly involved in xylan hydrolysis. The second protein contained no recognizable catalytic GH domain while the third one harbored GH39 domain, which is found in xylan β-1,4-xylosidase, and likely performed the hydrolysis of (1,4)-β-D-xylans, to remove successive D-xylose residues from the non-reducing termini. Eight other GH39 enzymes carrying N-terminal signal peptides encoded in the SH-KS-3 genome could also perform this function in xylan hydrolysis. One of these enzymes additionally contained two GH5 domains and could act as a multifunctional endo-β-1,4-xylanase/xylan β-1,4-xylosidase. 

Besides xylanases, enzymes enabling the cleavage of side chains of xylans were identified, including α-L-arabinofuranosidase and acetyl xylan esterase. The ability to utilize xylose was consistent with the presence of the isomerase pathway of xylose metabolism, including xylose isomerase and xylulose kinase. The phosphoketolase pathway of xylose utilization was not encoded as evidenced by the absence of its key enzyme, xylulose-5-phosphate phosphoketolase.

The analysis of the SH-KS-3 genome predicted the ability of this bacterium to utilize other carbohydrates. Besides xylan hydrolysis, secreted GH5 and GH8 enzymes could be responsible for the extracellular hydrolysis of cellulose, while the intracellular hydrolysis of produced cellobiose could be performed by GH94 family cellobiose phosphorylases. The genome also encoded transporters and utilization pathways for various simple sugars, including arabinose, ribose, mannose, maltose and maltodextrin, fructose, lactose, galactose, and N-acetylglucosamine. The glycerol utilization pathway, including glycerol uptake facilitator, glycerol kinase, and glycerol-3-phosphate dehydrogenase were also present.

Particularly notable was the presence of the whole pathway for L-rhamnose and L-fucose metabolism. The genome contained 15 genes for α-L-rhamnosidase that could cleave terminal non-reducing alpha-L-rhamnose residues from α-L-rhamnosides. Nine of these enzymes also contained CBM67 domain specific to this substrate [51]. The cleavage of L-fucose could be performed by α-L-fucosidases. L-rhamnose imported into the cell via L-rhamnose/proton symporters was converted into L-rhamnulose by isomerase; then, it was phosphorylated by rhamnulokinase and cleaved by aldolase into dihydroxyacetone phosphate and lactaldehyde. The metabolism of L-fucose followed a similar pathway. Dihydroxyacetone phosphate directly entered the Embden–Meyerhof glycolytic pathway while the subsequent metabolism of lactaldehyde to lactate proceeded in metabolosomes, microcompartments bounded by a proteinaceous shell and designed to metabolize compounds toxic to the cell [52]. A cluster of genes encoding structural proteins of the metabolosome was found in the SH-KS-3 genome. Lactate could be converted to pyruvate by L-lactate dehydrogenase encoded at the same locus. The oxidation of L-lactate to pyruvate could also be enabled by a membrane-associated LutABC lactate dehydrogenase complex encoded by an operon comprising a rhamnulokinase gene. Metabolosomes involved in the utilization of L-rhamnose and L-fucose have previously been identified in some *Planctomycetota, Verrucomicrobiota* and *Acidobacteriota* [53,54,55].

### 3.4. Central Metabolic Pathways of Chthoniobacteraceae SH-KS-3 Bacterium

The analysis of the SH-KS-3 genome revealed key pathways for anaerobic heterotrophic metabolism, including the Embden–Meyerhof pathway, gluconeogenesis, the non-oxidative stage of the pentose phosphate pathway, and the tricarboxylic acid cycle. Pyruvate generated in glycolysis could be converted to acetyl–CoA via the pyruvate dehydrogenase complex, or to lactate by lactate dehydrogenase, or to formate and acetyl-CoA by pyruvate formate lyase. 

Acetyl–CoA could be converted to acetate with the concomitant production of ATP through a two-step reaction involving phosphate acetyltransferase and acetate kinase. Therefore, formate, acetate, and lactate are the probable products of fermentation. The lack of hydrogenases indicated that hydrogen could not be produced as a result of fermentative metabolism.

The major components of the electron transfer chain for energy generation via oxidative phosphorylation are encoded in the SH-KS-3 genome, namely the proton-translocating NADH:quinone oxidoreductase, membrane-bound succinate dehydrogenase, and terminal oxidases. The generated transmembrane proton gradient may be used for ATP synthesis via an F_0_F_1_-type ATPase. The anaerobic lifestyle of SH-KS-3 bacterium is indicated by the absence of the cytochrome *c* oxidases primarily responsible for aerobic respiration. Cytochrome *bd* oxidase was present; such enzymes have a high affinity for oxygen [56] and can be involved in respiration and oxygen detoxification when the bacterium enters microaerobic ecological niches. Protection against reactive oxygen species can also be enabled by superoxide dismutase and catalase encoded in the genome and typically found in aerobes rather than in strict anaerobes.

A genome analysis revealed limited capacities of SH-KS-3 bacterium for anaerobic respiration. Membrane-linked NapG-type periplasmic nitrate reductase could enable nitrate reduction to nitrite, but reductases for the subsequent reduction in nitrite were not found. Known reductases for anaerobic respiration with sulfate and other sulfur compounds, arsenate, and iron were not found.

The SH-KS-3 genome encodes multiple mechanisms enabling the assimilation of nitrogen. Ammonium uptake can be performed by three AmtB family ammonium transporters. Urea is another source of nitrogen since the genome contained a gene cluster with genes for urea carboxylase, allophanate hydrolase, urea carboxylase-related aminomethyltransferase, and urea carboxylase-related ABC transporter. Another pathway of urea utilization, based on urease, was missing. The presence of N-acetylglucosamine transporters and genes enabling the conversion of N-acetylglucosamine into fructose-6-phosphate with the release of ammonium suggested that N-acetylglucosamine may be used as a nitrogen source. Ethanolamine could be another organic nitrogen source as indicated by the presence of ethanolamine ammonia–lyase, which can generate acetaldehyde and NH_3_. In addition to ammonium and organic nitrogen sources, the SH-KS-3 bacterium has the capacity to fix atmospheric N_2_. The SH-KS-3 genome contains three gene clusters encoding iron–iron, molybdenum–iron, and vanadium–iron nitrogenases. Interestingly, two of them seemed to have been laterally acquired since close homologs of genes for the iron–iron nitrogenase were found, not in *Verrucomicrobiota*, but in *Methanobacteriota* and *Firmicutes*, and vanadium–iron nitrogenase has the closest homologues in *Cyanobacteria* (*Nostoc* sp.).

### 3.5. Comparative Genomics of Chthoniobacteraceae

**Figure 4:**
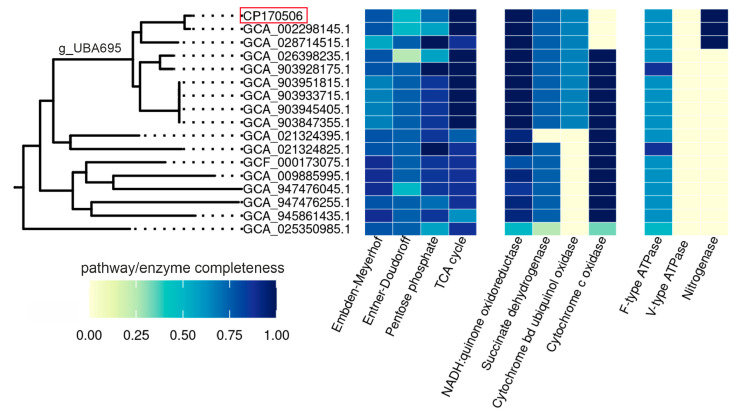
Comparative analysis of the members of *Chthoniobacteraceae* using the DRAM tool.

At present, in addition to the genus *Chthoniobacter*, the family *Chthoniobacteraceae* comprises eight uncultivated candidate genera defined on the basis of the MAGs (Figure 2). In order to gain insight into the metabolic capacities of *Chthoniobacteraceae*, we performed a comparative analysis of the metabolic pathways present in the representatives of all these genera using the DRAM tool (Figure 4). Since only the SH-KS-3 and *Chthoniobacter flavus* genomes were complete and assembled as the closed chromosomes, the absence of particular genes or the incompleteness of pathways in other members of the *Chthoniobacteraceae* should be interpreted with caution as it may have resulted from the incompleteness of the corresponding MAGs.

All the members of *Chthoniobacteraceae* seemed to have the Embden–Meyerhof glycolytic pathway, the non-oxidative stage of the pentose phosphate pathway, and the TCA cycle. The Wood–Ljungdahl pathway and the Calvin cycle for autotrophic carbon fixation were missing in all the MAGs.

Aerobic respiratory chains with terminal cytochrome *c* oxidases were found in most *Chthoniobacteraceae* genomes except for SH-KS-3 and the phylogenetically close MAGs, UBA695 and STA_34. All of them belong to the genus UBA695, but other species of this genus harbor cytochrome *c* oxidases. Therefore, anaerobic lifestyle is not typical for *Chthoniobacteraceae*. Only SH-KS-3, UBA695, and STA_34 bacteria have nitrogenase for nitrogen fixation. It is probable that the loss of cytochrome c oxidase and the acquisition of nitrogenase reflected an adaptation of this group of species to anaerobic lifestyle. 

Some members of *Chthoniobacteraceae* possess genes enabling anaerobic respiration with nitrogen compounds, but their sets differ among the genomes and a complete denitrification pathway is not encoded in any of them. None of the genomes encoded dissimilatory sulfate reduction pathways and reductases for other sulfur compounds.

### 3.6. Description of Candidatus Chthoniomicrobium xylanophilum

The SH-KS-3 MAG is the first complete genome in the candidate genus, UBA695, of the family *Chthoniobacteraceae*, recognized in the GTDB taxonomy. Since it is a finished MAG [57], we propose the following taxonomic names:

Description of “*Candidatus* Chthoniomicrobium” gen. nov: [Chtho.ni.o.mi.cro′bi.um. Gr. adj. *chthonios*, born from the soil, also the name of one of the Spartoi of the Cadmus myth; N.L. neut. n. *microbium*, from Gr. adj. *mikros* small and Gr. n. *bios* life, a microbe; N.L. neut. n. *Chthoniomicrobium,* microbe from the soil];

Description of “*Candidatus* Chthoniomicrobium xylanophilum” sp. Nov: [*xylanophilum* (xy. la. no’ phi. lum. Gr. n. *xylon*, wood; M. L. n. *xylanum*, xylan; Gr. adj. *philos*. liking, friendly to; N. L. neut. adj., *xylanophilum*. liking xylan].

This was not cultivated and was inferred to be an anaerobic mesophile, obligate organotroph utilizing xylan. It was inferred to be able to utilize cellulose and low-molecular-weight organic substrates, including rhamnose, fucose, arabinose, ribose, mannose, maltose, fructose, lactose, galactose, N-acetylglucosamine, and glycerol. It was predicted to be able to fix atmospheric nitrogen and grow by fermentation or anaerobic respiration with nitrate. The cells were predicted to be capable of twitching motility. They were represented by the complete genome assembled from the metagenome of the anaerobic enrichment culture on xylan, obtained from the peat sample from the Shichengskoe fen, Vologda Region, Russian Federation.

### 3.7. Xylanolytic Capacities of Other Bacteria in Xylan-Supplied Enrichment Culture

The genera *Bacillus* and *Clostridium* comprise many species well-known for their ability to hydrolyze complex plant polysaccharides including xylan (reviewed in [58,59,60,61]). However, xylanolytic capacity was not reported for *Bacillus wiedmannii*, and the inspection of the SH-KS-12 genome did not reveal genes for the utilization of xylan and xylose. Therefore, SH-KS-12 bacterium could not act as a primary xylanolytic and likely utilized low molecular weight organics produced by other community members.

In contrast, the *Clostridium* SH-KS-8 genome encoded the “true” endo-1,4-β-xylanase of the GH11 family. Other encoded enzymes involved in xylan utilization were the beta-xylosidases of the GH39 and GH43 families, α-1,2-glucuronidase, and α-L-arabinofuranosidase. The xylose transporters and enzymes involved for their intracellular processing were encoded as well, suggesting that SH-KS-8 is able to breakdown and utilize xylose.

*Caproiciproducens* sp. are known as strictly anaerobic bacteria capable of the fermentation of sugars and production of medium-chain fatty acids such as caproate via chain elongation (reverse-*β* oxidation) [62]. However, the ability to hydrolyze xylan was not reported for this genus. An analysis of the SH-KS-11 genome did not reveal genes encoding potential endoxylanases, although xylose transporters and enzymes for the intracellular metabolism of xylose via isomerase pathway were encoded. Probably, *Caproiciproducens* SH-KS-11 took advantage of the availability of xylose produced by primary xylanolytic microorganisms.

*Cellulomonas* species are known to be capable of degrading various polysaccharides, such as starch, xylan, and cellulose, as well as crystalline cellulose [63]. These bacteria possess a complex set of extracellular glucanases, some of which are multifunctional and have a multi-domain structure. Considering potential endoxylanases, SH-KS-1 genome annotation predicted a single candidate: glycoside hydrolase containing a N-terminal secretion signal peptide, a GH30 catalytic module, and a CBM6 substrate-binding domain. According to the CAZY database, different activities were described for the GH30 enzymes, including endo-β-1,4-xylanase and xylan β-1,4-xylosidase, while CBM6 can bind to cellulose and xylan. The genes for xylan 1,4-β-xylosidase (GH39 family), α-L-arabinofuranosidases, and acetyl xylan esterases, as well as xylose isomerase and xylulose kinase, were also predicted in the SH-KS-1 genome. Therefore, *Cellulomonas* SH-KS-1 could be involved in xylan utilization, although it seems unlikely that this bacterium plays the role of a primary xylan-degrader in enrichment cultures.

The genus *Rhizomicrobium* of the *Alphaproteobacteria* comprises facultatively anaerobic fermentative bacteria typically found in soils associated with plant roots [64]. The SH-KS-5 genome comprises a distinct region harboring genes for xylan hydrolysis and utilization, including two GH10 family endo-1,4-β-xylanases, xylan 1,4-β-xylosidase of the GH52 family, two α-D-glucuronidases, xylose isomerase, and xylulose kinase. Genes encoding other enzymes involved in xylan hydrolysis (α-L-arabinofuranosidase etc.) were also identified in the genome. Therefore, the SH-KS-5 bacterium could be directly involved in xylan hydrolysis.

Since the enrichment cultures on xylan were obtained under anaerobic conditions, we checked the presence of the genetic determinants of aerobic respiration in the assembled MAGs. Cytochrome *c* oxidases were identified in the *Bacillus* SH-KS-12, *Cellulomonas* SH-KS-1 and *Rhizomicrobium* SH-KS-5 genomes, but were absent in *Clostridium* SH-KS-8 and *Caproiciproducens* SH-KS-11.

## 4. Discussion

The requirement of a complex set of enzymes for the complete hydrolysis of xylan suggests the participation of a consortium of microorganisms specializing in individual stages. The abilities of pure microbial strains to efficiently degrade hemicelluloses are generally limited to relatively simple substrates, such as artificial xylan. Several studies show that the consortia of different microorganisms can degrade natural lignocelluloses more efficiently than its members [65,66]. For example, artificial communities consisting of *Bacillus* and *Clostridium* strains showed enhanced extracellular xylanase activity, and a higher lignocelluloses degradation capability than any of the pure cultures [65].

To characterize the microorganisms capable of hydrolyzing xylan under the anaerobic conditions typical of wetland soils, we obtained anaerobic enrichment cultures on xylan and analyzed their composition and the genetic potential of its members. The obtained cultures contained several bacterial species. Interestingly, the same OTUs were selected in independent cultures inoculated with peat samples from two fens located at a distance of about 160 km from each other, which indicated the similarity of the xylanolytic community’s composition in different wetlands with similar physical and chemical characteristics. Moreover, successive incubations with xylan did not result in the selection of monocultures of xylan degraders, which confirms the importance of the synergistic action of different microorganisms.

Metagenomic analysis of the obtained cultures revealed that besides representatives of the phylum *Firmicutes*, which together with *Bacteroidota* usually play a key role in the degradation of lignocellulose, the consortium contains an organism from the *Chthoniobacteraceae* family of the phylum *Verrucomicrobiota*, and this bacterium makes up more than half of the consortium. Analysis of the MAGs of the consortium members revealed a probable division of their functions in the utilization of xylan.

The dominant bacterium, *Chthoniobacteraceae* SH-KS-3 possess a set of secreted xylan β-1,4-xylosidases some of which also contain xylan-specific CBM9 domain. In the same time, among enzymes of the GH10 and GH11 families, known to comprise active endo-1,4-β-xylanases [21], only one GH10 family hydrolase was found. Endoxylanase activity was also found for GH5 and GH8 family enzymes and several extracellular hydrolases of these families were predicted in the SH-KS-3 genome. Considering enzymes enabling cleavage of side chains of xylans, α-L-arabinofuranosidase and acetyl xylan esterase were identified, while α-D-glucuronidase of the GH67 family was not found. Therefore, *Chthoniobacteraceae* SH-KS-3 could possess a near complete set of enzymes required for xylan hydrolysis, although the functions of potential endoxylanases need to be experimentally evaluated.

*Clostridium* SH-KS-8 and *Rhizomicrobium* SH-KS-5 are most likely candidates for the role of primary xylan degraders. The first bacterium encodes endo-1,4-β-xylanase of the GH11 family, and the second harbors a distinct xylan utilization locus with two genes of GH10 family endo-1,4-β-xylanases. Other enzymes, required for cleavage of side groups, processing of xylo-oligosaccharides, import of xylose and its intracellular metabolism are also encoded. Other community members probably take an advantage of the availability of xylo-oligosaccharides and xylose produced by primary xylan degraders (*Cellulomonas* SH-KS-1 and *Caproiciproducens* SH-KS-11) or utilizes low molecular weight organics (*Bacillus* SH-KS-12).

The finding of *Chthoniobacteraceae* as the dominant phylotype in enrichment on xylan was rather unexpected since *Verrucomicrobiota* are not regarded as highly efficient xylan degraders. Nevertheless, there are several reports demonstrating their involvement in xylan hydrolysis. A combination of fluorescently labeled substrates, fluorescence-activated cell sorting, and single cell genomics was applied to analyze freshwater and coastal bacterioplankton for degraders of laminarin and xylan [67]. This study revealed Verrucomicrobiota cells attached to xylan in single-cell sorting experiments and their genomes encoded a wide range of glycoside hydrolases [67]. The subsequent study showed that verrucomicrobia are key players in laminarin and xylan hydrolysis in an arctic fjord of Svalbard [68]. The analysis of MAG from the Baltic Sea assigned to verrucomicrobial subdivision 2 revealed glycoside hydrolases that likely allow the use of a variety of carbohydrates, like cellulose, mannan, xylan, chitin, and starch, as carbon sources [69]. Verrucomicrobial MAGs with the predicted capacity of xylan, chitin, or cellulose degradation were obtained from freshwater reservoirs [70]. Recently, the growth on xylan was observed for a facultatively anaerobic chemoorganoheterotrophic bacterium, *Fontisphaera persica,* representing the order *Limisphaerales* [71]. At present, the family *Chthoniobacteraceae* contains a single cultured member, *C. flavus* Ellin428 [26]. This strain can utilize a wide range of carbohydrates and is able to grow aerobically with simple sugars (glucose, fructose, galactose, mannose, xylose, cellobiose, lactose, sucrose), and various polysaccharides, xylan, starch, cellulose, and pectin [26]. Therefore, the ability to utilize xylan may be a common trait in *Chthoniobacteraceae*, although the adaptation to an anaerobic lifestyle in nitrogen-poor environments seems to be limited to SH-KS-3 bacterium and its closest relatives.

## 5. Conclusions

❖ The laboratory incubation of peat samples with xylan under anaerobic conditions resulted in the pronounced enrichment of several phylotypes of *Verrucomicrobiota, Firmicutes,* and *Alphaproteobacteria*;
❖ The enrichment culture was dominated by the uncultured genus-level lineage of the family, *Chthoniobacteraceae,* of the phylum, *Verrucomicrobiota*;
❖ The complete closed genome of this bacterium, designated “*Candidatus* Chthoniomicrobium xylanophilum” SH-KS-3, was assembled;
❖ An analysis of the SH-KS-3 genome revealed potential endo-1,4-β-xylanases, as well as xylan β-1,4-xylosidases and other enzymes involved in xylan utilization. The SH-KS-3 bacterium also possessed enzymes enabling the utilization of cellulose, various simple sugars, and glycerol;
❖ A genome analysis showed the absence of aerobic respiration and suggested a chemoheterotrophic lifestyle through fermentation or nitrate reduction;
❖ An analysis of other MAGs obtained from the metagenome of the enrichment cultures suggested that *Clostridium* and *Rhizomicrobium* could play the role of primary xylan degraders while other community members probably take advantage of the availability of the xylo-oligosaccharides and xylose produced by primary xylan degraders or utilize low molecular weight organics;
❖ Indigenous microbial communities of peat fens have a high potential for polysaccharide degradation.

## Figures and Tables

**Table 1:** Physical and chemical properties of peat water at the sampling sites.

Characteristic	Shichengskoe Fen	Charozerskoe Fen
EC (μS/cm)	408	286
pH	7.4	7.0
Organic C (%)	73.6	66.2
N total (%)	2.31	2.4
SO_4_^2−^ (mg/L)	202	188
Fe (ppm)	9387	5333
Ca (ppm)	29,834	31,193
Mg (ppm)	2575	2695
P (ppm)	1179	985

**Table 2:** Main characteristics of MAGs obtained in this work.

Bin Id	Completeness/Contamination (%)	Contigs	MAG Size (bp)	Fraction of Metagenome (%)	Taxonomy (GTDB)
SH-KS-3	97.97/0.68	1 *	5,624,388	58.5	p__Verrucomicrobiota; c__Verrucomicrobiae; o__Chthoniobacterales; f__Chthoniobacteraceae; g__UBA695
SH-KS-11	97.15/1.01	1 *	3,914,149	3.1	p__Firmicutes_A; c__Clostridia; o__Oscillospirales; f__Acutalibacteraceae; g__Caproiciproducens
SH-KS-12	98.95/0.18	3	5,507,092	3.7	p__Firmicutes; c__Bacilli; o__Bacillales; f__Bacillaceae_G; g__Bacillus_A; s__Bacillus_A wiedmannii
SH-KS-8	96.33/2.47	1 *	6,087,521	1.2	p__Firmicutes_A; c__Clostridia; o__Clostridiales; f__Clostridiaceae; g__Clostridium
SH-KS-5	98.99/0.8	22	5,624,037	3.1	p__Proteobacteria; c__Alphaproteobacteria; o__Micropepsales; f__Micropepsaceae; g__Rhizomicrobium
SH-KS-1	92.2/0.39	10	4,151,991	2.2	p__Actinobacteriota; c__Actinomycetia; o__Actinomycetales; f__Cellulomonadaceae; g__Cellulomonas

* closed circular genome.

## Data Availability

The complete genome sequence of the “*Candidatus* Chthoniomicrobium xylanophilum” strain SH-KS-3 has been deposited in the NCBI GenBank database under the accession number CP170506.
